# A Neurodynamic Account of Spontaneous Behaviour

**DOI:** 10.1371/journal.pcbi.1002221

**Published:** 2011-10-20

**Authors:** Jun Namikawa, Ryunosuke Nishimoto, Jun Tani

**Affiliations:** Brain Science Institute, RIKEN, Wako, Japan; Indiana University, United States of America

## Abstract

The current article suggests that deterministic chaos self-organized in cortical dynamics could be responsible for the generation of spontaneous action sequences. Recently, various psychological observations have suggested that humans and primates can learn to extract statistical structures hidden in perceptual sequences experienced during active environmental interactions. Although it has been suggested that such statistical structures involve chunking or compositional primitives, their neuronal implementations in brains have not yet been clarified. Therefore, to reconstruct the phenomena, synthetic neuro-robotics experiments were conducted by using a neural network model, which is characterized by a generative model with intentional states and its multiple timescales dynamics. The experimental results showed that the robot successfully learned to imitate tutored behavioral sequence patterns by extracting the underlying transition probability among primitive actions. An analysis revealed that a set of primitive action patterns was embedded in the fast dynamics part, and the chaotic dynamics of spontaneously sequencing these action primitive patterns was structured in the slow dynamics part, provided that the timescale was adequately set for each part. It was also shown that self-organization of this type of functional hierarchy ensured robust action generation by the robot in its interactions with a noisy environment. This article discusses the correspondence of the synthetic experiments with the known hierarchy of the prefrontal cortex, the supplementary motor area, and the primary motor cortex for action generation. We speculate that deterministic dynamical structures organized in the prefrontal cortex could be essential because they can account for the generation of both intentional behaviors of fixed action sequences and spontaneous behaviors of pseudo-stochastic action sequences by the same mechanism.

## Author Summary

Various psychological observations have suggested that the spontaneously generated behaviors of humans reflect statistical structures extracted via perceptual learning of everyday practices and experiences while interacting with the world. Although those studies have further suggested that such acquired statistical structures use chunking, which generates a variety of complex actions recognized in compositional manner, the underlying neural mechanism has not been clarified. The current neuro-robotics study presents a model prediction for the mechanism and an evaluation of the model through physically grounded experiments on action imitation learning. The model features learning of a mapping from intentional states to action sequences based on multiple timescales dynamics characteristics. The experimental results suggest that deterministic chaos self-organized in the slower timescale part of the network dynamics is responsible for generating spontaneous transitions among primitive actions by reflecting the extracted statistical structures. The robustness of action generation in a noisy physical environment is preserved. These results agree with other neuroscience evidence of the hierarchical organization in the cortex for voluntary actions. Finally, as presented in a discussion of the results, the deterministic cortical dynamics are presumed crucial in generating not only more intentional fixed action sequences but also less intentional spontaneously transitive action sequences.

## Introduction

Our everyday actions are full of spontaneity. For example, imagine that a man makes a cup of instant coffee every morning. After he pours hot water into his mug, which is already filled with a spoonful of coffee crystals, he may either add sugar first and next add milk, or add milk first and then add sugar. Or, sometimes he may even forget about adding sugar and notice it later when he first tastes the cup of coffee. Some parts of these action sequences are definite, but other parts are varied because we see spontaneity in the action generation. Similar actions can be seen in improvisations in playing jazz or in contemporary dance, where musical phrases or body movement patterns are inspired freely from one to another in an unpredicted manner. An essential question is from where the spontaneity for generating voluntary actions or images originates. The current article presents a model prediction for the underlying neural mechanism.

We speculate that the necessary neural structures for generating such spontaneous actions are acquired as the results of learning from everyday experiences and practices while interacting with the world. Gibson and Pick [1] once wrote that infants are active learners who perceptually engage their environments and extract information from them. In their ecological approach, learning an action is not just about learning a motor command sequence. Rather, it involves learning the possible perceptual structures extracted during intentional interactions with the environment. Other researchers [2]–[4] have proposed that perceptual structures experienced during environmental interactions can be acquired by using forward models that are assumed to be located in the cerebellum. A forward model outputs the prediction of the next sensation by receiving the inputs of the current sensation and motor commands. Although this idea is generic and theoretical in the sense that a forward model can predict the sensory outcomes of arbitrary motor commands at every time step, it is impossible in practice due to the combinatorial explosion problem if the motor dimension becomes large. This problem is analogous to the frame problem [5] that discusses that there are no rational means to stop inferences about the outcomes of infinite action possibilities.

In humans, learning by environmental interaction does not proceed with all possible combinations in motor command sequences, but with more purposeful or intentional stances [1]. Therefore, it could be sufficient for humans to predict sensory outcomes according only to purposeful behavior trajectories. With regard to this theory, Tani [6] proposed an alternative idea that an agent should learn mapping from a set of particular intentional states with the consequent sensory sequences that are expected to be experienced in corresponding purposeful environmental interactions. Ay et al. [7] also proposed a prediction based model to control an autonomous robot, but the model lacked internal states. In the connectionist implementation by Tani and his colleagues [6], [8], [9], the proposed neuro-dynamic model is operated in three modes: generation, recognition and learning. In the generation mode, the corresponding visuo-proprioceptive sequence is predicted for a given intentional state. The scheme can generate mental simulation for future behaviors or motor imagery (in terms of visuo-proprioceptive sequences) [10], and can also generate the corresponding physical movement by sending the next predicted proprioceptive state to the motor controller as the next target. In the recognition mode, a given visuo-proprioceptive sequence can be recognized by identifying the corresponding intentional state through an iterative search to minimize the prediction error. In the learning mode, the learning is formulated as a process to search for the optimal values for both a common synaptic weight matrix as well as a set of individual intentional states that can regenerate all visuo-proprioceptive sequences of an experience for training under minimum error criteria. This idea is formally related to “active inference” [11], which can be regarded as a form of predictive coding [12]. Friston [13] showed that the three aspects of our neurodynamic model (generation, recognition and learning) can be unified in terms of minimising prediction error.

Moreover, it is presumed that the neural structures acquired through intentional interactions with the environment should support “compositionality” [14] or chunk knowledge for generating and recognizing the variety of complex actions [15]–[17]. Diverse intentional actions can be generated by combining a set of reusable behavior primitives or chunks adaptively by following the acquired rules. For example, an attempt at drinking a cup of water can be decomposed into multiple behavior primitives, such as reaching for a cup, grasping the cup and moving the cup toward one's mouth. Each behavior primitive can be re-used as a component for other intentional actions, e.g., reaching for a cup to take it away. Psychological observation on infant development as well as adult learning have suggested that chunking structures in perceptual streams can be extracted by statistical learning with sufficient amounts of passive perceptual experiences [18], [19] as well as for active behavioral interactions [20]. Here, chunking structures are represented by repeatable sensory sequences within chunks and the probabilistic state transition among those chunks. From his observation of skill acquisitions for food processing by mountain gorillas, Byrne [21] proposed that actions can be acquired with statistical structures through imitation. It is said that juvenile gorillas take a few years to effectively imitate behaviors by observing the mothers' food processing behaviors, which are characterized by nondeterministic transition sequences of behavior primitives or chunks. After the skill acquisition by extracting the underlying statistical structures, the primitive sequences of the juveniles resemble those of their mothers. In another example, improvisers of jazz music make substantial efforts into developing vocabularies of musical patterns or phrases, which they then freely combine and vary in a manner that is sensitive to the on-going musical context [22]. In recent years, considerable evidence has been assembled in support of statistical learning for both musical pitch sequences [23] and rhythm [24] by organizing chunking structures.

In searching for the neuronal mechanisms for chunking, a monkey electrophysiological study [25] showed that some neurons in the presupplementary motor area (preSMA) fire only at the beginning of each chunk in the regeneration of trained sequences. Also, a human behavior study [26] showed that inactivation of the preSMA by transcranial magnetic stimulation (TMS) affects the performance of regenerating sequences only when the TMS is applied between the chunks after extensive learning of the sequences with chunking structures. These studies suggest that the preSMA might play a crucial role in segmenting sequences into chunks. Sakai et al. [27] proposed a hierarchical function in a cortical network, in which the prefrontal cortex and the preSMA were responsible for the cognitive control of segmenting sequences and selecting the next chunk, whereas more motor-related areas, including the premotor cortex and the primary motor cortex, are responsible for processing within each chunk. This proposal agrees with the results of a monkey electrophysiological recording [28], [29], which showed that firing some cells in the preSMA and the SMA encode specific sequences of joystick movement patterns or specific transitions from one movement pattern to another.

Model studies by the authors have shown that chunking by segmenting continuous sensory flow can be achieved by applying the criteria of prediction error minimization to connectionist models with local [17], [30] and distributed [6], [31] representation schemes. Those model studies have also shown that a hierarchy is indispensable in acquiring chunking structures. In the hierarchy, the lower level learns to acquire a set of behavior primitives and the higher level puts those primitives into sequences of intentional actions. The idea of intentional actions is analogous to that by Sakai et al. [32].

One essential question so far is how the next behavior primitives or actions can be decided by following the learned statistical expectation. For example, if we suppose that someone has learned that the next behavior primitive to use is either primitive-A or primitive-B, given an even chance from past experiences, it is plausible to consider that either the primitives can be decided by her/his conscious will or alternatively they can be determined automatically without consciousness. Philosophers have discussed for a long time whether humans have a “free will” to determine a next action arbitrarily, and, if the free will exists, how it is implemented in our minds [33]–[35]. Interestingly, recent neuropsychological studies on free decision [36], [37] have suggested that the conscious will of initiating or selecting actions arbitrarily follows certain neural activity that does not accompany awareness. Libet et al. [36] showed that a conscious decision to press a button was preceded for a few hundred milliseconds by a negative brain potential, referred to as the readiness potential, which originates from the SMA. In a functional magnetic resonance imaging (fMRI) study, Soon et al. [37] demonstrated that brain activity is initiated in the prefrontal cortex (PFC) and the parietal cortex up to seven seconds before a conscious decision. It was further found that the observed brain activity can predict the outcome of a motor decision, which the subject did not consciously make, such as pushing the left button by the left index finger or the right button by the right index finger. This result implies the possibility that even though someone can believe that he had consciously decided a particular action among multiple choices, such as primitive-A and primitive-B, the conscious decision was not the direct cause of the action selection, but was the preceded neural activity without awareness. It is also plausible to assume a purely mechanist model in which an itinerant trajectory of neural dynamics [38]–[41], instead of a conscious will, determines the next behavior primitive to be performed.

The current article introduces a neuro-robotics experiment that examines how statistical structures hidden in skilled behaviors can be extracted via imitation learning and how the behaviors can be regenerated by following the statistical structures. The experiment uses a dynamic neural network model based on two essential ideas from our previous proposals. First, in the dynamic neural network model, a mapping from the initial states of the internal neural dynamics to the expected visuo-proprioceptive trajectories is self-organized, and the initial states encode various intentional states for the resultant behavioral trajectories [9]. Second, the model network employs multiple timescale dynamics [31], [42] that allow self-organization of the functional hierarchy, which is analogous to the known cortical hierarchical network consisting of the PFC, the preSMA, and the M1 [43], [44] for generating goal-directed actions.

It is demonstrated that itinerant behaviors with accompanying spontaneous transitions among behavior primitives can be generated by reflecting the observed statistical structure and by assuring the robustness of the behavior primitives against possible noise during physical interactions, when deterministic chaos is self-organized at the higher level of the functional hierarchy (the slow dynamics part of the network). Based on the results, the current article discusses the importance of deterministic neural dynamics in action generation because they account for both itinerant behaviors with accompanying spontaneous transitions of behavior primitives and intentional fixed behaviors (repeatedly executable) by the same dynamic mechanism. The article also discusses how the model prediction presented can be applied in future neurophysiological studies.

## Results

### Model Overview

**Figure 1:**
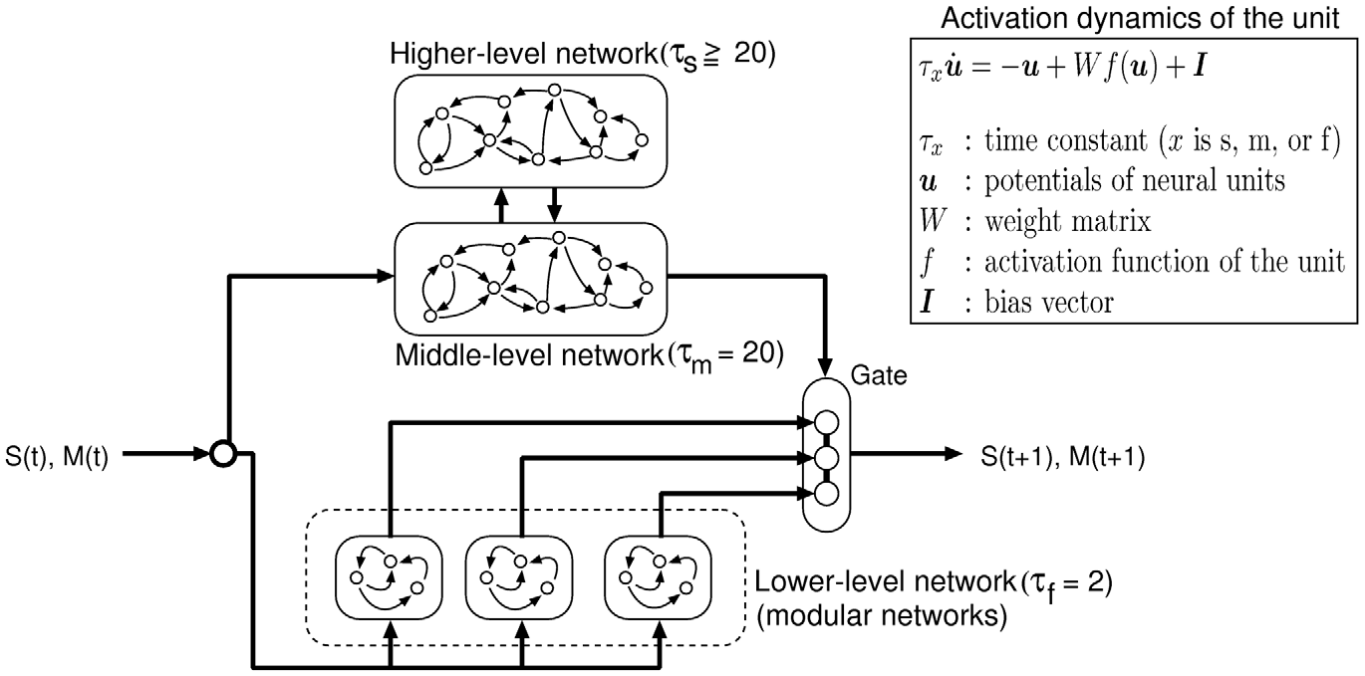
Hierarchical neural network consisting of three levels characterized by time constants of the neural activation dynamics. The higher-level, middle-level, and lower-level networks consist of neural units in which the activations are characterized by large (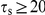), moderate (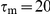), and small (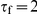) time constants, respectively. The visuo-proprioceptive input 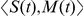 for each time 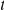 reaches the middle- and lower-level networks, and the middle level relays the input to the higher level. The lower-level network contains a set of modular networks generating motor commands, which, in turn, are forwarded to the gate. The gate, which prevents undesired motor commands from being released, is controlled by the middle-level network. Since the supplementary motor area (SMA) has been reported to trigger the movement by suppressing the inhibitory signal exerted on the primary motor cortex [61], the middle-level and lower-level networks may correspond to the SMA and the primary motor cortex, respectively. The higher-level network may correspond to the prefrontal cortex (PFC), which projects to the SMA [62].

The proposed hierarchical dynamic neural network can be regarded as a generative model of visuo-proprioceptive inputs [6], [11], [31], [45]. (Precise mathematical descriptions are provided in the Methods section.) The network was divided into three levels based on the value of the time constant 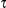. Time constant 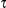 for each unit primarily determined the timescale of the activation dynamics of the unit (see Figure 1). The higher level consisted of slow neural units with a larger time constant (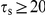), the middle level with a moderate time constant (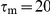), and the lower level with a small time constant (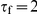). The lower level was assembled with a set of gated modular networks [46] that interacted directly with the visuo-proprioceptive sequences. The higher level was mutually connected to the middle level but was not directly connected to the lower level. The middle level interacted with the lower level by sending the gate opening signals and receiving the sensory inputs.

The parameters of this model were optimized to minimize sensory prediction errors (i.e., maximizing the probability of the predictions given the sensory data). In this sense, our model was concerned with, and only with, perception. Action per se, was a result of movements that conformed to the proprioceptive predictions of the joint angles. This means that perception and action were both trying to minimize prediction errors throughout the hierarchy, where movement minimized the prediction errors at the level of proprioceptive sensations. With this perspective, the high-level network provided predictions of the middle-level network that, in turn, provided predictions about which expert will be engaged for prediction at the lowest (sensory) level. This engagement depended upon the gating variables, which selected the most appropriate expert in the lowest level of the hierarchy.

The network was trained to predict a set of given visuo-proprioceptive sequences by optimizing the following two types of parameters in order to minimize the prediction error: the synaptic weights and the initial state of the internal units in the whole network for each sequence [9]. This intuitively means that the learning involves determining the dynamic function of the network by changing the synaptic weights and also by inferring the intention or goal of each action sequence. The learning scheme was implemented by using the error back-propagation through time algorithm [47]. Although the biological plausibility of error back-propagation in neuronal circuits is a matter of debate, some supportive evidence [48], [49] and related discussions [50] exist. We speculate that the retrograde axonal signal [50] conveying the error information might propagate from the sensory periphery area to the higher-order cortical area by passing through multiple stages of synapses and neurons for modulating the intentional states.

After minimizing the error, each visuo-proprioceptive sequence of the training can be regenerated by setting the initial state of the internal units with the optimized value. Because the initial state specified the expected visuo-proprioceptive sequence, the initial states are considered to represent the intentions of generating specific actions. The forward dynamics of the trained network can generate motor imagery in terms of visuo-proprioceptive sequences by feeding back the sensory prediction computed at the previous time step into the current sensory inputs without the accompanying physical movements (closed-loop operation). However, the physical movements can be generated by sending the next time step prediction of the proprioceptive states (joint angles) to the motor controller as the target (open-loop operation).

As one aspect of our work, we examine how the dynamic function of each level can be differentiated depending on the timescale differences. To study the timescale characteristics in more detail, we investigated cases applying various values of the time constant 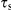 set in the higher-level network.

### Design of Robot Experiments

**Figure 2:**
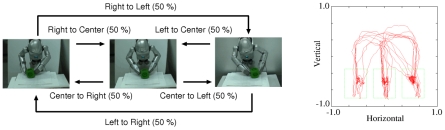
Schematic diagram of the teaching sequence generation process. As shown on the left, the assistant selected one action randomly while guiding the hands of the robot to move the cubic object. For each action, the assistant started from a posture in which the hands of the robot were open and then the assistant grasped the object by using the hands of the robot. Whenever the object was moved to another position, the assistant released the object and returned the robot hands to the starting posture for a brief period of time. The graph on the right depicts the trajectory of the center of the object captured by the vision sensor.

Experiments on imitation learning of actions were conducted on a small humanoid robot platform (Sony Corporation), and the movie of a demonstration is available (http://www.bdc.brain.riken.jp/tani/mov/PLoS11.html). The robot experiments on the aforementioned dynamic neural network model involved imitative training of the sequences of primitive actions and autonomous generation of those imitated behaviors. The target primitive action sequences to be imitated were designed with a statistical structure and with transitioning of the primitive actions, and the sequences were directly tutored to the robot, i.e., a human assistant directly guided the movements of both hands of the robot by grasping them. In the beginning of each training sequence, the assistant guided both hands of the robot, which was positioned in front of a workbench (see Figure 2). A cubic object was placed on the workbench at one of three positions (center, 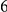 cm left of center, and 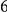 cm right of center), and the assistance repeated primitive actions of grasping the object, moving it to one of the three positions and releasing it by guiding the hands of the robot while deciding the next object position randomly with equal probability (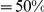). Although the hands of the robot were guided by the assistant, the visuo-proprioceptive sequences were recorded for later training. The neural network was trained in an off-line manner, since all training sequences gathered at each teaching session were used for the subsequent consolidation learning. Thus, the neural network learned to predict visuo-proprioceptive sequences on the basis of the experiences obtained during the imitative training session. Note that no explicit cues for segmenting the sequences into primitive actions were prepared. The related chunking structures were acquired via iterative experiences of the continuous visuo-proprioceptive sequences.

After the imitative training, each training sequence was regenerated by setting the initial state with the optimized value. For the subsequent analysis, primitive action sequences were generated and recorded for far longer than the training sequences. For the detailed analysis on the possible dynamic structures acquired in the network model, the itinerant trajectories by motor imagery for longer steps were also generated.

### Training and Action Generation

The network was trained by a learning scheme in which the higher-level time constant 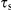 was 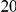, 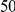, and 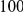 [45] (see the Methods section for details). For each condition of the higher-level time constant, training trials were conducted for 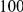 sample networks with different initial parameters and training data. Descriptions of the learning parameters are provided in the Methods section. The trained networks were tested for pseudo-generation by motor imagery, and it was shown that the networks regenerated primitive action sequences pseudo-stochastically in their deterministic itinerant trajectories. Here, pseudo-stochasticity denotes stochasticity observed through the discretization of deterministic continuous value sensory sequences into symbolically labeled primitive action sequences. The action generation test by motor imagery, also described in the Methods section, revealed that the trained neural networks were able to create novel sequential combinations of the primitive actions that were not contained in all teaching sequences. This implies that primitive actions can be generated pseudo-stochastically, as taught in all 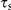 conditions.

We also tested cases of training primitive action sequences having different probabilities of selected primitive actions in a specific object position, as detailed in Text S1. It was observed that these probabilities were reconstructed in the generation of the primitive action sequences by motor imagery. The results suggest that the network is capable of extracting underlying statistical structures in the imitated primitive action sequences.

Next, we tested the generation of actual actions of interacting with the physical environment by the robot. First, the behavior of the robot was generated from each acquired initial state with the higher-level time constant, 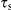 set at 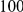. Although it was observed that the trained primitive action sequences can be regenerated exactly during the initial period (7.3 primitive action transitions on average), the sequences gradually became different from the trained ones. It was considered that this result was due to the initial sensitivity characteristics organized in the trained network.

**Figure 3:**
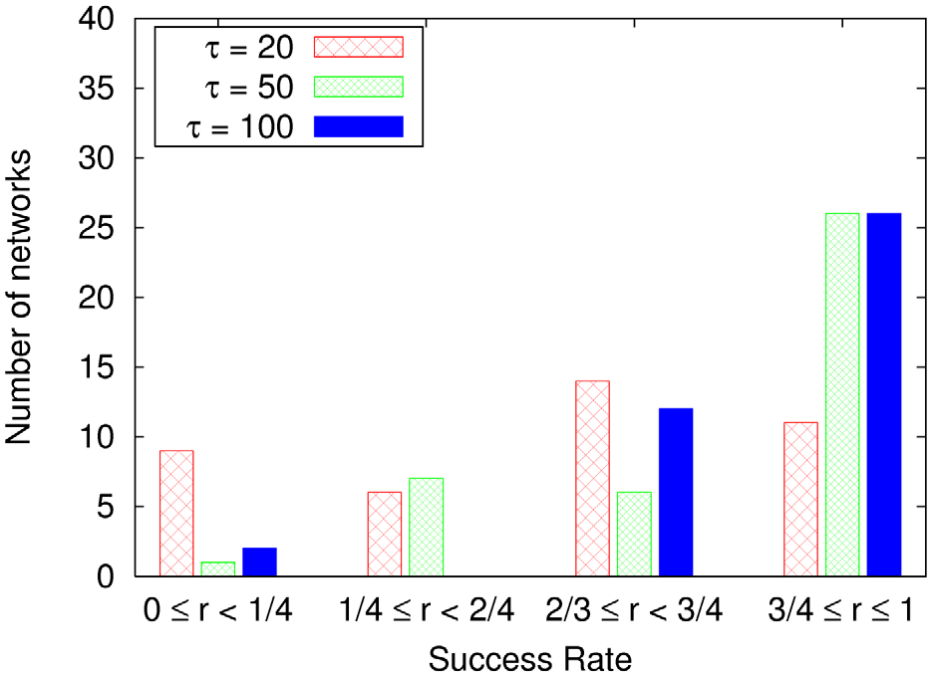
Success rate 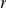 of a robot controlled by a neural network to move an object. For example, 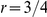 indicates 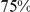 success of moving the object without dropping it. The graph depicts the frequencies of networks occurring in certain ranges of the success rate. We recorded the behaviors of the robot for up to 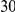 trials of moving the object for 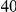 sample networks. The average success rates for the higher-level time constant 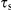 of 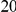, 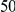, and 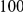 were 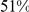, 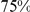, and 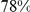, respectively.

Then, we tested actual action generations for cases of different values of time constant 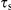. Although no particular difference was found between the cases with a different 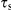 in the action generation test by the motor imagery mode, the stability in actual action generation in the physical environment was different for each 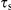. The success rate for moving the object without dropping it was evaluated for each trained network with a different time constant 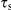. Figure 3 shows the frequencies of the networks classified according to the success rate, where populations of 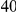 individual networks were trained for each time constant 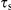. In all cases we found a network with a 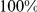 success rate, but the average success rate was different for each 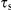. The success rate for 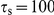 was higher than the success rates for the other values of 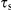.

This result indicates that motor patterns can be generated stably in the physical environment when 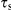 is set larger than 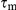.

### Self-Organized Functional Hierarchy

**Figure 4:**
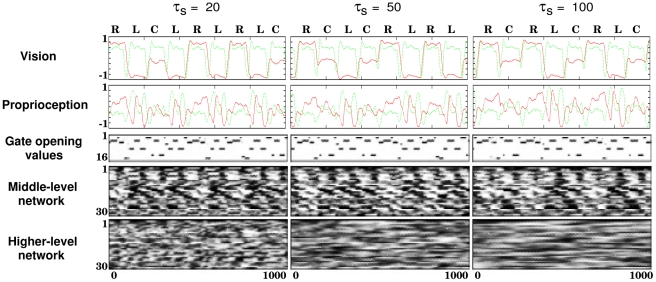
Time series generated by a trained network (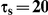, 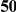, and 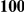). In the vision case, the two lines correspond to the relative position of the object (red: horizontal, green: vertical). Each label over the vision sensor values denotes the object position (L: left, C: center, and R: right). In proprioception, 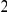 out of a total of 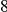 dimensions were plotted (red: left arm pronation, green: left shoulder flexion). The lower three figures show the time series described by grayscale plots, where the vertical axis represents the indices of the neural units. A long sideways rectangle thus indicates the activity of a single unit over many time steps.

As mentioned in the previous section, the stability in actual action generation is dependent on the timescale characteristics. This fact implies that the developed dynamic structure is different for each timescale condition. In the following we discuss the characteristics of the self-organized functional hierarchy in terms of the timescale differences. As an example, Figure 4 illustrates the sensory motor sequences and neural states. It can be seen that an action primitive of moving the object to the left, to the right, or to the center consisted of a few different gate openings, which generated sequential switching of the stored reusable motor patterns, such as reaching for the object, picking up the object, and moving back its hand position to the starting posture. It is considered that the middle- and higher-level network dynamics encoded the combinations of these reusable motor patterns into primitive actions and further into their stochastically switching sequences.

**Figure 5:**
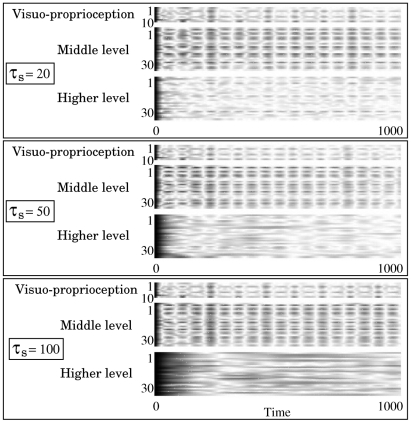
Auto-correlation for each neural unit, shown by grayscale plots. The vertical axes denote the indices of the neural units, and the horizontal axes denote the length of time.

The auto-correlation for each 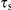 is shown in Figure 5 to clarify the timescale characteristics. The auto-correlation measures the correlation between values at different points in time and is sometimes used to find repeating patterns. A high auto-correlation at time difference 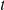 means that similar values appear repeatedly with a period of 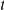. We found a periodic pattern of auto-correlation common to both the visuo-proprioception and the middle-level network units in the 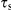 cases. This periodicity is considered to occur because the periodicities of all primitive actions were approximately the same. Conversely, such a periodic pattern was not found in the higher-level network if the higher-level network had a relatively large time constant. The characteristics of auto-correlation seem to correspond to the functionality obtained by each subnetwork.

**Figure 6:**
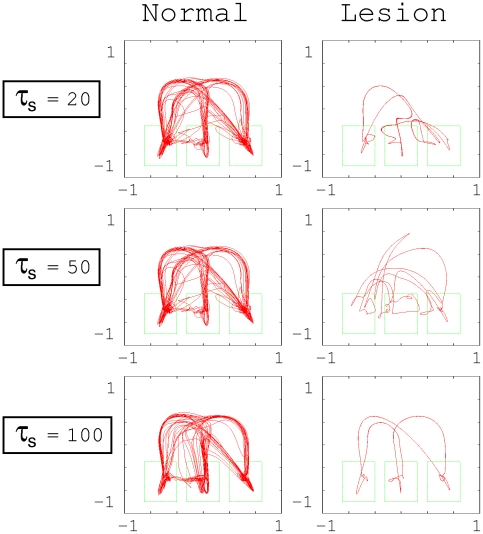
Trajectory of the object position captured by the vision sensor in the motor imagery mode. Left: normal case, Right: removal of the higher-level network case (lesion case).

To examine the functionality developed at each subnetwork, we investigated the effect of artificial lesions in the higher-level network. To model the artificial lesions, we removed all the neurons in the higher-level network. Figure 6 is a comparison of the trajectory of the object position captured by the vision sensor in the motor imagery mode for the normal case and that with the artificial lesions. In this figure, we used networks having a 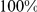 success rate in actual action generation. If the trajectory generated by the network traced the test data (see Figure 2), the network moved the object by correctly using the hands. When the time constant of the higher level was set as 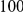, the network was able to generate each single primitive action correctly, even if the higher level was removed. However, in this case, the capability for combining diverse primitive actions significantly deteriorated. For 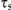 set at 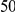 or 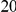, the removal of the higher level significantly affected the generation of each primitive action. This implies that the functions for generating each primitive action and for generating stochastic combinations of these actions were self-organized and became segregated between the higher and the middle/low levels if 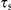 was set significantly larger than 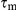 and 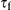. Otherwise, both functions were self-organized but not segregated. That is, the functions were distributed throughout the entire network. In this situation, a lesion in the higher-level network could affect the lower sensory-motor control level. The abovementioned functional segregation between levels could contribute to the stability in the action performance evaluated in Figure 3.

In addition, the maximum Lyapunov exponents for the subnetworks were computed while varying 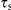 as 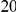, 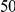, and 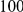 (see Methods for details). Here, 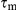 and 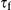 were fixed at 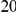 and 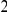, respectively. The computation was repeated for 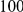 neural networks trained using different initial synaptic weights but under the same learning condition. If the Lyapunov exponent is found to be positive for specific subnetworks or for the entire network, then the intrinsic dynamics of the subnetworks or network are identified as chaotic. In chaotic dynamics, almost any minute change in an internal neural state brings about a drastic change in subsequent network outputs because of the dependence on the initial conditions. Therefore, it can be inferred that subnetworks having positive maximum Lyapunov exponents act to combine action primitives with pseudo-stochasticity. The results are summarized in Table 1. The maximum Lyapunov exponent of the entire network was positive for all values of 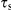. This was expected because the network was able to generate pseudo-stochastic primitive action sequences regardless of the value of 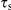. However, the values of the maximum Lyapunov exponent were negative in the higher level and the middle level, when the higher-level network had a small time constant 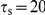. These results indicate that the function to generate chaos was globally distributed over the entire network. In contrast, when the higher-level network was set with a large time constant, i.e., 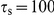, the maximum Lyapunov exponent of the higher-level network became positive in most cases (94 out of 100 network learning cases), whereas that of the middle-level network became negative. The abovementioned results demonstrate that if the time constant of the higher level is sufficiently larger than the time constants of the other regions, then chaotic dynamics are formed primarily in the higher-level network, separate from the other regions. These results agree with the results in the case having lesions. Furthermore, the abovementioned analysis on the artificial lesion cases indicates that the segregation of chaos from the middle and lower levels provides more stable motor generation. In summary, if we regard our neurodynamic model as a generative model of visuo-proprioceptive sequences, the anatomical segregation between sequential dynamics and motor primitives (e.g., within premotor and motor cortex, respectively) emerges only when we accommodate the separation of temporal scales implicit in the hierarchical composition of those sequences.

**Table 1:** Maximum Lyapunov exponents of various regions (mean of 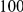 sample networks).

Time constant 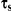 of the higher level	Entire network	Middle-level network	Higher-level network
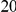	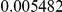 ( 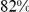 )	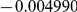 ( 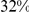 )	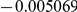 ( 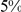 )
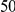	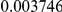 ( 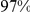 )	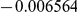 ( 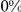 )	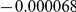 ( 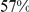 )
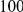	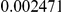 ( 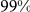 )	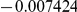 ( 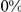 )	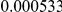 ( 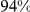 )

The percentages in parentheses represent the probability that the maximum Lyapunov exponent of each network was positive in multiple training trials.

Finally, we examined how the generation of chaos that allows pseudo-stochastic transition among action primitives depends on the characteristics of the training sets. For this purpose, the networks with 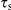 set at 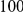 were trained by changing the length (i.e., number of transitions of primitive actions) of the training sequences, as detailed in Text S1. It was observed that the possibility of generating chaos is reduced as the length of the training sequences is reduced. If no chaos was generated, it was observed that neural activity in the higher-level network often converged to a fixed point some steps after the tutored sequences were regenerated. This result implies that the mechanism for spontaneous transition by chaos can be acquired only through training of long sequences that contain statistically enough probabilistic transitions for generalization.

## Discussion

### Spontaneous Action Generation by Chaos

The current experimental results revealed that the chaos self-organized in the higher-level network with slow-timescale dynamics facilitates spontaneous transitions among primitive actions by following statistical structures extracted from the set of visuo-proprioceptive sequences imitated, whereas primitive actions were generated in the faster-timescale networks in the lower level. The finding was repeatable in the robotic experiments, provided that the timescale differences were set adequately among the different levels of subnetworks. The results appear to be consistent with human fMRI recordings, which indicate that free-decision-related activity without consciousness is slowly built up in the PFC seconds before the conscious decision [37]. This buildup of activity in the PFC could initiate a sharp response in the SMA just a few hundred milliseconds before the decision [36]. Activation in the SMA leads to immediate motor activity [28], and buildup of action-related cell activity in the PFC in the monkey brain takes a few seconds, whereas that in the primary motor area takes only a fraction of a second [51]. These observations support recent arguments concerning the possible hierarchy along the rostro-caudal axis of the frontal lobe. Badre and D'Esposito [43] proposed that levels of abstraction might decrease gradually from the prefrontal cortex (PFC) through the premotor cortex (PMC) to the primary motor cortex (M1) along the rostro-caudal axis in the frontal cortex in both the monkey brain and the human brain [44]. Here, the rostral part is considered to be more integral in processing information than the caudal part in terms of its slower timescale dynamics. By considering the possible roles of the M1, such as encoding the posture or direction of limbs [52], [53], it is speculated that this hierarchy in the frontal cortex contributes to the predictive coding of proprioceptive sequences through the M1 in one direction, and to that of visual sequences in the other direction via the possible connection between the inferior parietal cortex and the SMA, known as the parieto-frontal circuits [54], [55].

Additionally, our experiments showed that the behavior generation of the robot in the real environment becomes substantially unstable when the timescale of the higher-level network is set smaller, that is, when the timescale is similar to the values in the middle-level network. Our analysis in such cases revealed that the two functions of generating primitive motor patterns and sequencing them cannot be segregated in the whole network if the chaos dynamics tend to be distributed. Gros [56], who referred to higher-level as the reservoir and to the middle/low levels as attractor networks, also discussed the generation and stabilization of transient state dynamics, in terms of attractor ruins. The discussion supports one's opinion that the slower timescale part exhibits robustness of influence of external stimuli. Therefore, it is concluded that the hierarchical timescale differences, which are assumed to be in the human frontal cortex and to be responsible for the generation of voluntary actions, are essential for achieving the two functions of freely combining actions in a compositional manner and generating them stably in a physical environment.

### Deterministic versus Stochastic Process

The uniqueness in the presented model is that deterministic chaos is self-organized in the process of imitating stochastic sequences, provided that sufficient training sequences are utilized to support the generalization in learning (it was observed that the reduction of length in the training sequences can hinder the self-organization of chaos). Therefore, it might be asked why deterministic dynamical system models are considered more crucial than stochastic process models such as the Markov Chain [57] or Langevin equation [58], [59]. A fundamental reason for focussing on deterministic (as opposed to stochastic) dynamics is that they allow for mean field approximations to neuronal dynamics and motor kinetics. This is important because although individual neuronal dynamics may be stochastic, Fokker Planck formulations and related mean field treatments render the dynamics deterministic again; and these deterministic treatments predominate in the theoretical and modelling literature. Furthermore, we speculate that deterministic neuronal dynamic systems are indispensable for generating both spontaneous behaviors and intentional behaviors under the same dynamic mechanism, especially by applying the initial sensitivity characteristics. When the system is initiated from unspecified initial states of the internal units, the resultant itinerant behavioral trajectories exhibit spontaneous transitions of primitive actions by reflecting the statistical structures extracted through the generalization in learning. However, it is also true that a particular sequence of shorter length can be regenerated by resetting with the corresponding initial state values by the deterministic nature of the model. Our robotics experiments showed that the robot can regenerate trained sequences up to several transitions of primitive actions under a noisy physical environment. In addition, our preliminary experiments suggested that longer primitive action sequences can be stably regenerated if those intentional sequences are trained more frequently than other non-intentional ones. This implies that frequently activated fixed sequences can be remembered by cash memories of their initial states. If the Markov chain model is employed for reconstructing the same feature, the model must handle the dualistic representation, namely the probabilistic state transition graph for generating itinerant behaviors and the deterministic linear sequence for generating each intentional behavior.

Empirical support for the idea of encoding action sequences by initial states can been found. Tanji and Shima [28] found that some cells in the SMA and the preSMA fire during the preparatory period immediately before generating specific primitive action sequences in the electrophysiological recording of monkeys. The results can be interpreted such that those cell firings during the preparatory period may represent the initial states that determine which primitive action sequences to be generated subsequently. In the future, if this study can be extended to simultaneous observations of the populations of animal cells during spontaneous action sequence generation by employing the recent developments of multiple-electrode recording techniques, our model prediction can be further evaluated.

## Methods

### Model Implementation

The evolution of a continuous-time-rate coding model is defined as
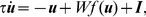
(1)where 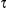 is a time constant, 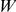 is a weight matrix, 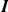 is a bias vector, and 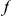 is the activation function of a unit (typically the sigmoid function or 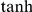). When this differential equation is put into the form of an approximate difference equation with step size 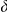, we obtain
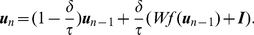
(2)In the present paper, we assume 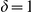 without the loss of generality.

As the hierarchical neural network for controlling a humanoid robot, we used a mixture of the recurrent neural network (RNN) experts model [45], in which the gating network is a multiple-timescale RNN [31]. The mixture of RNN experts consists of expert networks together with the gating network. All experts receive the same input and have the same number of output units. The gating network receives the previous gate opening values and the input, and then controls the gate opening. The role of each expert is to compute a specific input-output function, and the role of the gating network is to decide which single expert is the winner on each occasion. In the present paper, the set of expert RNNs is referred to as the lower-level network. The middle- and higher-level networks differentiated by time constants are contained in the gating network.

The dynamic states of the lowest-level neural networks (the mixture of experts) at time 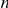 are updated according to
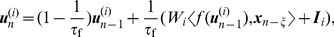
(3)

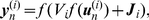
(4)

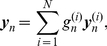
(5)where 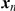 is an input vector representing the current visuo-proprioception, and 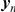 is an output vector representing the predicted visuo-proprioception. Here, 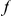 denotes a component-wise application of 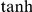, 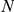 is the number of experts, and 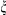 is the feedback time delay of the controlled robot (in the experiment 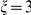). In the present paper, 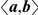 denotes the concatenation of vectors 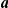 and 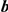. For each 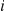, the terms 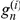, 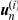, and 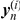 denote the gate opening value, the internal neural state, and the output state of the expert network 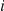, respectively. The gate opening vector 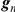 represents the winner-take-all competition among experts to determine the output 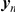. The gate opening vector 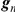 is the output of the gating network, defined by
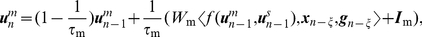
(6)

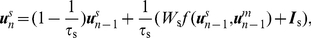
(7)

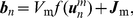
(8)

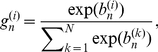
(9)where 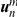 and 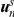 denote the internal neural states of the middle-level and higher-level networks, respectively. To satisfy 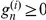 and 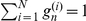, Equation (9) is given by the soft-max function. Using the sigmoid function denoted by 
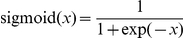
, Equation (9) can be expressed as
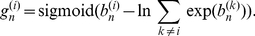
(10)This equation indicates that the output of the gating network is given by the sigmoid function with global suppression. Note that the visuo-proprioceptive input 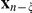 enters the state Equations (3) and (6). During imagery, this is replaced by its prediction 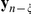 so that these equations become (in discrete form) nonlinear autoregression equations that embody the learned dynamics. During inference and action, optimising the parameters of these equations can be thought of as making them into generalised Bayesian filters such that the predictions become maximum a posterior predictions under the (formal) priors on the dynamics specified by the form of these equations.

### Learning Method

The procedure for training the hierarchical neural network model is organized into the following two phases:

1. We first train the experts (lower level) by optimizing their parameters. Crucially, in this phase, the gating variables are treated as unknown parameters and are optimized according to the teaching sensory data.
2. We then train the gating networks (middle and higher levels) by optimizing their parameters to predict the gating variables optimized in Phase (1).

The training procedure progresses to phase (2) after the convergence of phase (1). For each phase, the learning involves choosing the best parameter based on the maximum a posteriori estimation. A learning algorithm with a likelihood function and a prior distribution was proposed in [45]. In the following, we describe a learning algorithm corresponding to the above description of the hierarchical neural network model.

#### Probability distribution

To define the learning method, we assign a probability distribution to the hierarchical neural network. Here, the adjustable parameters of an expert network 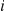 and a gating network are denoted as 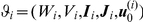 and 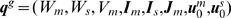, respectively. The estimate 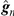 of the gate opening vector is given in terms of the variable 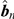, as follows:
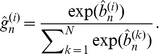
(11)


Let 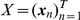 be an input sequence of visuo-proprioception, and let 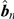 and 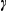 be parameters, where 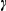 is a set of parameters given by 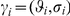. Given 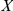, 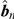, and 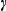, the probability density function (p.d.f.) for the output 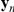 is defined by
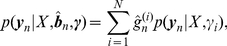
(12)where 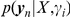 is given by
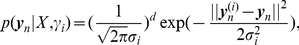
(13)where 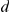 is the output dimension, and 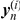 is the output from expert 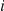, as computed by Equations (3), (4), and (5) with the parameter 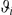. Thus, the output of the model is governed by a mixture of normal distributions. These equations result from the assumption that the observable sequence data is embedded in additive Gaussian noise. Minimizing the mean square error is equivalent to maximizing the likelihood determined by a normal distribution for learning in a single neural network. Therefore, Equation (13) is a natural extension of neural network learning.

Given a parameter set 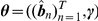 and an input sequence 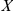, the probability of an output sequence 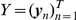 is given by
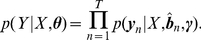
(14)The function 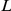 of data set 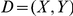 parameterized by 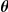 is defined by the product of the likelihood 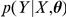 and a prior distribution, denoted as
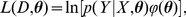
(15)where 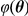 is the p.d.f. of a prior distribution given by
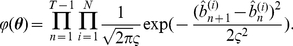
(16)This equation indicates that the vector 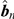 is governed by 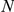-dimensional Brownian motion. The prior distribution has the effect of suppressing changes of the gate opening values. We can now optimize the unknown parameters in Equation (15) with respect to function 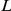. These parameters include the variables underlying the optimum gating, the connection strengths of the lower level, and the parameters controlling the variance of sensory noise. We next consider Phase 2, in which the parameters of the gating network are optimized.

Assume that 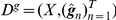 is given. We define the likelihood 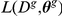 by the multinomial distribution
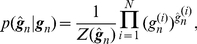
(17)

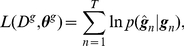
(18)where 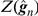 is the normalization constant, and 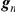 is given by 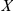 and 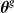. Note that 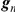 in Equation (18) is calculated by the teacher forcing technique [60], i.e., using 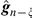 instead of 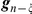 in Equation (6). In addition, because of the term 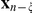 on the right-hand side of Equation (6), 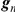 cannot be computed if 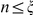. Then, we assume that 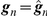 if 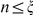. If we consider 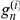 to be the probability of choosing an expert 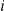 at time 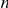, maximizing 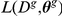 is equivalent to minimizing the Kullback-Leibler divergence
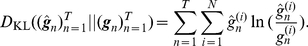
(19)


#### Maximum a posteriori estimation

The learning method chooses the best parameters 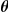 and 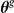 by maximizing (or integrating over) the likelihood 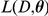 and 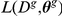. More precisely, we use the gradient descent method with a momentum term as the training procedure. The model parameters at learning step 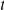 are updated according to
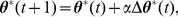
(20)

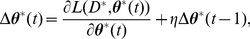
(21)where 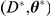 is 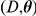 or 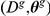. Here, 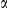 is the learning rate, and 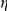 is the momentum term parameter.

Although we have explained only the case in which the training data set 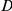 is a single sequence, the proposed method can easily be extended to the learning of several sequences by using the sum of the gradients for each sequence. When several sequences are used as training data, initial states 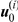, 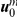, and 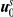 and an estimate 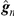 of the gate opening vector must be provided for each sequence.

#### Acceleration of gating network learning

In principle, the present training procedure is defined by Equations (21) and (20). However, the learning for a gating network sometimes becomes unstable, and so the likelihood 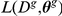 often decreases when updating the parameter 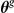 with certain learning rates [45]. Of course, if we use sufficiently small learning rates, the likelihood does not decrease for any learning step. However, considerable computational time is required. Hence, to practically accelerate gating network learning, we update the learning rate adaptively by the following algorithm.

1. For each learning step, updated parameters are computed by Equations (21) and (20) by applying the learning rate 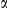, and the rate 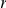 defined by
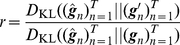
(22)is also computed, where 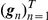 and 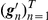 are sequences of the gate opening values corresponding to the current parameters and the updated parameters, respectively.

2. If 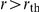, then 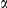 is replaced with 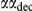, and return to (1). Otherwise, go to (3).

3. If 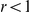, then 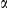 is replaced with 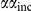. Go to the next learning step.

In the present study, we use 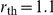, 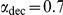, and 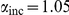.

### Parameter Settings for Training

In the training processes, we used 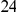 training sequences, including 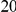 action primitives of moving an object to the left, to the center, and to the right. The time constants for the lower-level and middle-level networks were set to 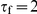 and 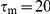, respectively. The time constant for the higher level was chosen to be 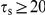. The number of experts was 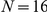, and the number of internal units for each expert was 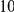. There were 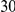 internal units for the middle-level network and 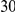 for the higher-level network, i.e., the total number of internal units in the gating network was 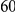. Each element of the matrices and biases of either an expert or a gating network was initialized by random selection from the uniform distribution on the interval 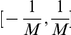, where 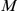 is the number of internal units. The initial states for the experts and the gating network were also initialized randomly from the interval 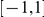. The parameters 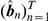 and 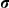 were initialized such that 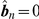 for each 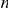 and 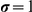, respectively. Since the maximum value of 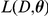 depends on the total length 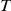 of the training sequences and the dimension 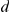 of the output units, the learning rate 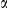 was scaled by a parameter 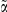 that satisfies 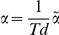. The parameter settings were 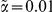, 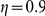, 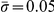, and 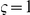.

For each training trial, we conducted learning for the experts up to 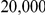 steps and learning for the gating network up to 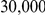 steps. We performed training of the experts for 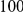 samples having different initial parameters and training data. In addition, for each set of trained experts, we performed training of the gating network while varying the higher-level time constant 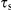 at 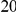, 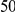, and 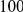. As a result, 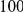 samples of a mixture of RNN experts were provided for each condition of the higher-level time constant.

### Robotic Platform

The behaviors of the robot were described by a 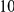-dimensional time series, which consists of proprioception (an eight-dimensional vector representing the angles of the arm joints) and vision sense (a two-dimensional vector representing the object position). On the basis of the visuo-proprioception, the neural network generated predictions of the proprioception and vision sense for the next time step. This prediction of the proprioception was sent to the robot in the form of target joint angles, which acted as motor commands for the robot to generate movements and interact with the physical environment. This process, in which values for the motor torque were computed from the desired state, was considered at a computational level to correspond to the inverse model. This inverse computation process was preprogrammed in the robot control system. Changes in the environment, including changes in the object position and changes in the actual positions of the joints, were sent back to the system as sensory feedback.

### Action Generation Test

**Figure 7:**
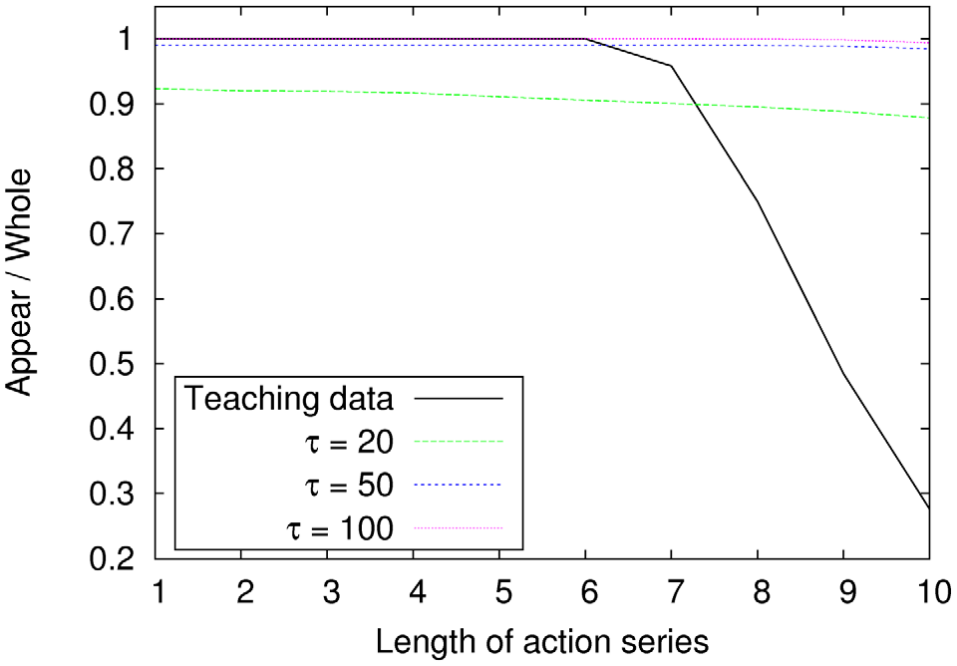
Performance for creating novel sequential combinations of primitive actions. This graph shows the ratio of the number of 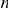-blocks generated by the network to the total number of possible 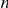-blocks. The total number of acceptable combinations (of length 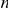 of the primitive action series) was 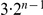. To evaluate the ratio, the data for 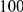 sample networks were averaged for each condition of the higher-level time constant 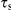 in a numerical simulation.

We demonstrate how a trained network learns to generate combinations of primitive actions. Let us consider a sequence of symbols labeled according to the object position, e.g., “CLRLRCL
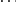
”, where C, L, and R are the center, left, and right positions, respectively. Let 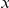 be a block if 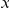 is a finite sequence of symbols. An 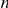-block is simply a block of length 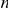. To evaluate the performance in creating novel sequential combinations, we counted the number of 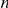-blocks that appeared in the visuo-proprioceptive time series generated by the network. We computed the ratio of the number of 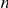-blocks generated by the network to the total number of possible 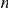-blocks (note that the total number of possible 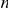-blocks is 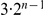). Figure 7 shows the ratios averaged over 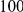 sample networks with higher-level time constants 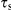 of 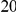, 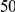, and 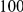 in the motor imagery mode. Note that the teaching sequences do not include all acceptable combinations, because the teaching data are finite.

### Maximum Lyapunov Exponent

The maximum Lyapunov exponent of a dynamic system is a quantity that characterizes the rate of exponential divergence from the perturbed initial conditions. Consider two points, 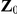 and 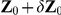, in a state space, each of which generates an orbit in the space by the dynamic system. The maximum Lyapunov exponent 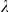 can be defined as
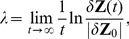
(23)where 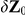 is the initial separation vector of two trajectories, and 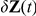 is the separation vector at time 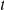.

To evaluate the maximum Lyapunov exponent for each neural network, we computed 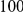 sample sequences of 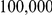 time steps with a random initial state and an initial separation vector by a numerical simulation. In the numerical simulation, a network received predictions of the visuo-proprioception generated by the network itself as input for the next step. When the maximum Lyapunov exponent of a middle- or higher-level network was measured, we computed the dynamics of the entire network, but evaluated a separation vector containing only the component of a subnetwork as a middle- or higher-level component. This method measures the contribution of the subnetwork to the initial sensitivity of the dynamics. Note that if the subnetwork has a positive Lyapunov exponent, as measured in the abovementioned manner, then the entire network also has a positive Lyapunov exponent.

## Supporting Information

Text S1 **Supporting online material for “A Neurodynamic Account of Spontaneous Behaviour.”** This file describes two types of additional experiment, namely “Reconstruction of probability” and “Dependency on training data length”. These experimental results clarify the possible relation between chaos generated and the condition of the statistical learning employed in the model network.
(PDF)

## References

[1] Gibson E, Pick A (2000). An ecological approach to perceptual learning and development..

[2] Ito M (1970). Neurophysiological aspects of the cerebellar motor control system.. Int J Neurol.

[3] Uno Y, Kawato M, Suzuki R (1989). Formation and control of optimal trajectory in human multi-joint arm movement.. Biol Cybern.

[4] Wolpert D, Ghahramani Z, Jordan M (1995). An internal model for sensorimotor integration.. Science.

[5] McCarthy J (1963). Situations, actions, and causal laws..

[6] Tani J (2003). Learning to generate articulated behavior through the bottom-up and the top-down interaction processes.. Neural Netw.

[7] Ay N, Bertschinger N, Der R, Güttler F, Olbrich E (2008). Predictive information and explorative behavior of autonomous robots.. Eur Phys J B.

[8] Tani J, Ito M, Sugita Y (2004). Self-organization of distributedly represented multiple behavior schemata in a mirror system: reviews of robot experiments using rnnpb.. Neural Netw.

[9] Nishimoto R, Namikawa J, Tani J (2008). Learning multiple goal-directed actions through self-organization of a dynamic neural network model: A humanoid robot experiment.. Adapt Behav.

[10] Jeannerod M (1994). The representing brain: Neural correlates of motor intention and imagery.. Behav Brain Sci.

[11] Friston K, Mattout J, Kilner J (2011). Action understanding and active inference.. Biol Cybern.

[12] Rao R, Ballard D (1999). Predictive coding in the visual cortex: a functional interpretation of some extra-classical receptive-field effects.. Nat Neurosci.

[13] Friston K (2010). The free-energy principle: a unified brain theory?. Nat Rev Neurosci.

[14] Evans G, Holdzman S, Leich C (1981). Semantic theory and tacit knowledge.. Wittgenstein: To follow a rule.

[15] Arbib M (1981). Perceptual structures and distributed motor control.. Handbook of Phys: The Nerv Syst II Motor Control.

[16] Sternad D, Schaal S (1999). Segmentation of endpoint trajectories does not imply segmented control.. Exp Brain Res.

[17] Tani J, Nolfi S (1999). Learning to perceive the world as articulated: an approach for hierarchical learning in sensory-motor systems.. Neural Netw.

[18] Saffran J, Aslin R, Newport E (1996). Statistical learning by 8-month-old infants.. Science.

[19] Kirkham N, Slemmer J, Johnson S (2002). Visual statistical learning in infancy: Evidence for a domain general learning mechanism.. Cognition.

[20] Baldwin D, Andersson A, Saffran J, Meyer M (2008). Segmenting dynamic human action via statistical structure.. Cognition.

[21] Byrne R (2003). Imitation as behaviour parsing.. Philos T Roy Soc B.

[22] Ashley R, Hallam S, Cross I, Thaut M (2009). Musical improvisation.. Oxford Handbook of Music Psychology.

[23] Saffran J, Johnson E, Aslin R, Newport E (1999). Statistical learning of tone sequences by human infants and adults.. Cognition.

[24] Desain P, Honing H, Sadakata M (2003). Predicting rhythm perception from rhythm production and score counts: The bayesian approach..

[25] Nakamura K, Sakai K, Hikosaka O (1998). Neuronal activity in medial frontal cortex during learning of sequential procedures.. J Neurophysiol.

[26] Kennerley S, Sakai K, Rushworth M (2004). Organization of action sequences and the role of the pre-sma.. J Neurophysiol.

[27] Sakai K, Hikosaka O, Miyauchi S, Takino R, Tamada T (1999). Neural representation of a rhythm depends on its interval ratio.. J Neurosci.

[28] Tanji J, Shima K (1994). Role for supplementary motor area cells in planning several movements ahead.. Nature.

[29] Shima K, Tanji J (2000). Neuronal activity in the supplementary and presupplementary motor areas for temporal organization of multiple movements.. J Neurophysiol.

[30] Namikawa J, Tani J (2008). A model for learning to segment temporal sequences, utilizing a mixture of RNN experts together with adaptive variance.. Neural Netw.

[31] Yamashita Y, Tani J (2008). Emergence of functional hierarchy in a multiple timescale neural network model: a humanoid robot experiment.. PLoS Comput Biol.

[32] Sakai K, Hikosaka O, Nakamura K (2004). Emergence of rhythm during motor learning.. Trends Cogn Sci.

[33] Hume D (1748/1977). An enquiry concerning human understanding.

[34] James W (1956). The Dilemma of Determinism, Reprinted in The Will to Believe.

[35] Dennett D (1984). Elbow room: The varieties of free will worth wanting.

[36] Libet B (1985). Unconscious cerebral initiative and the role of conscious will in voluntary action.. Behav Brain Sci.

[37] Soon C, Brass M, Heinze H, Haynes J (2008). Unconscious determinants of free decisions in the human brain.. Nat Neurosci.

[38] Tsuda I (1991). Chaotic itinerancy as a dynamical basis of hermeneutics in brain and mind.. World Futures.

[39] Skarda C, Freeman W (1987). How brains make chaos in order to make sense of the world.. Behav Brain Sci.

[40] Tani J (1998). An interpretation of the ‘self’ from the dynamical systems perspective: a constructivist approach.. J Conscious Stud.

[41] Friston K, Kiebel S (2009). Attractors in song.. New Math Nat Comput.

[42] Kiebel S, Daunizeau J, Friston K (2008). A hierarchy of time-scales and the brain.. PLoS Comput Biol.

[43] Badre D, D'Esposito M (2009). Is the rostro-caudal axis of the frontal lobe hierarchical?. Nat Rev Neurosci.

[44] Mita A, Mushiake H, Shima K, Matsuzaka Y, Tanji J (2009). Interval time coding by neurons in the presupplementary and supplementary motor areas.. Nat Neurosci.

[45] Namikawa J, Tani J (2010). Learning to imitate stochastic time series in a compositional way by chaos.. Neural Netw.

[46] Jacobs R, Jordan M, Nowlan S, Hinton G (1991). Adaptive mixtures of local experts.. Neural Comput.

[47] Rumelhart D, Hintont G, Williams R (1986). Learning representations by back-propagating errors.. Nature.

[48] Fitzsimonds R, Song H, Poo M (1997). Propagation of activity-dependent synaptic depression in simple neural networks.. Nature.

[49] Du J, Poo M (2004). Rapid bdnf-induced retrograde synaptic modification in a developing retino- tectal system.. Nature.

[50] Harris K (2008). Stability of the fittest: organizing learning through retroaxonal signals.. Trends Neurosci.

[51] Hoshi E, Shima K, Tanji J (2000). Neuronal activity in the primate prefrontal cortex in the process of motor selection based on two behavioral rules.. J Neurophysiol.

[52] Georgopoulos A, Kalaska J, Caminiti R, Massey J (1982). On the relations between the direction of two-dimensional arm movements and cell discharge in primate motor cortex.. J Neurosci.

[53] Graziano M, Taylor C, Moore T (2002). Complex movements evoked by microstimulation of pre- central cortex.. Neuron.

[54] Wise S, Boussaoud D, Johnson P, Caminiti R (1997). Premotor and parietal cortex: Corticocortical connectivity and combinatorial computations 1.. Annu Rev Neurosci.

[55] Rizzolatti G, Luppino G, Matelli M (1998). The organization of the cortical motor system: new concepts.. Electroen Clin Neuro.

[56] Gros C (2009). Cognitive computation with autonomously active neural networks: an emerging field.. Cogn Comput.

[57] Markov A (1971). Extension of the limit theorems of probability theory to a sum of variables connected in a chain.. Dynam Probabilist Syst.

[58] Langevin P (1908). On the theory of brownian motion.. CR Acad Sci (Paris).

[59] Grabert H (1982). Projection operator techniques in nonequilibrium statistical mechanics.

[60] Williams R, Zipser D (1989). A learning algorithm for continually running fully recurrent neural networks.. Neural Comput.

[61] Ball T, Schreiber A, Feige B, Wagner M, Lücking C (1999). The role of higher-order motor areas in voluntary movement as revealed by high-resolution EEG and fMRI.. Neuroimage.

[62] Selemon L, Goldman-Rakic P (1988). Common cortical and subcortical targets of the dorsolateral prefrontal and posterior parietal cortices in the rhesus monkey: evidence for a distributed neural network subserving spatially guided behavior.. J Neurosci.

