# Supplementary material for: A Neurodynamic Account of Spontaneous Behaviour
Source: PLoS Comput Biol. 2011 Oct 20;7(10):e1002221. doi: 10.1371/journal.pcbi.1002221 (PMC3197631; doi:10.1371/journal.pcbi.1002221)
Supplement: Text S1 — Supporting online material for “A Neurodynamic Account of Spontaneous Behaviour.” This file describes two types of additional experiment, namely “Reconstruction of probability” and “Dependency on training data length”. These experimental results clarify the possible relation between chaos generated and the condition of the statistical learning employed in the model network. (PDF) [file pcbi.1002221.s001.pdf]

Text S1: Supporting online material for "A Neurodynamic Account of Spontaneous Behaviour"

Jun Namikawa[1], Ryunosuke Nishimoto[1], Jun Tani[1,*]
1 Brain Science Institute, RIKEN, 2-1 Hirosawa, Wako-shi, Saitama 351-0198, Japan
∗ E-mail: tani@brain.riken.jp

Reconstruction of probability

We tested cases of training primitive action sequences with different probabilities of selecting primitive actions in a specific object position. In this testing experiment, the probability of selecting "Right to Center" (when object is located in the "Right" position) was varied among 12.5%, 25%, and 50% (i.e., "Right to Left" was varied among 87.5%, 75%, and 50%; see Figure 2 in the article), and other probabilities were fixed with 50% as the same as the previous experiment. For each condition of training sequences, training trials were conducted for 100 sample networks when higher-level time constants τ_s was 100 (other parameters settings for training were the same as the previous experiment). Figure 1 in Text S1 describes the probabilities evaluated from primitive action sequences generated by trained networks. The result confirms that the model network can extract probabilities of selecting primitive actions in training sequences and also can generate pseudo-stochastic sequences with respect to the probabilities.

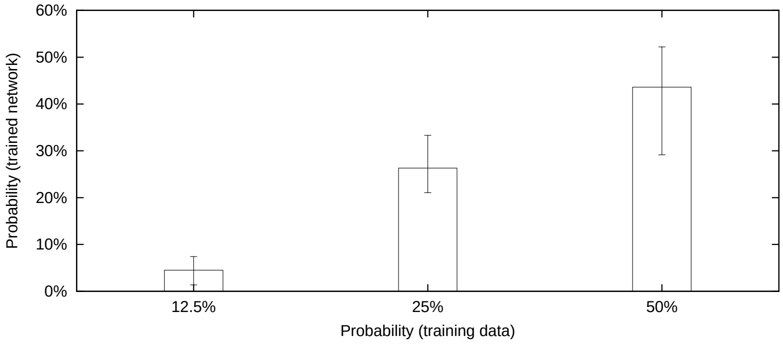

Figure 1. The results of training (mean of 100 training trials). Here, a probability refers to a probability of selecting action "Right to Center" in the right object position. When we evaluated the probability, we computed 50 sample sequences of $100,000$ time steps in the motor imagery mode for each network.

Dependency on training data length

We examined conditions of training sets for obtaining chaos by learning. On this purpose, we conducted an additional experiment in which the networks with τ_s set as 100 were trained by changing length of the training sequences. For each sequence in training sets, times of transitions of primitive actions were varied among 5, 10 and 20 (Note that the condition of 20 times was the same as the previous experiment). Other conditions for training were the same as the previous experiment. In Table 1 in Text S1, we present evaluated results of 100 sample networks in terms of probability of generating positive Lyapunov exponent value for each condition. It can be seen that generating chaos with positive Lyapunov exponent tends to require a longer length of training data. Figure 2 in Text S1 shows an example of visuo-proprioceptive sequences and internal neural activities for each condition. In the case of shorter length (Times=5), the

trajectory of internal neural activity in the higher-level network converged to a fixed point which resulted in repetition of a periodic cycle in the middle-level network, the gating values and the visuo-proprioceptive sequences. On the other hand in the case of longer length (Time=10, 20), pseudo-stochastic sequences were observed in the sequences.

Table 1. The percentage of networks whose maximum Lyapunov exponent was positive after training (by means of 100 sample networks). When we evaluated the maximum Lyapunov exponent, we computed 10 sample sequences of 1000, 000 time steps for each network.

Times of transitions in each training sequence	Percentage of networks whose Lyapunov exponent is positive
20	99%
10	82%
5	58%

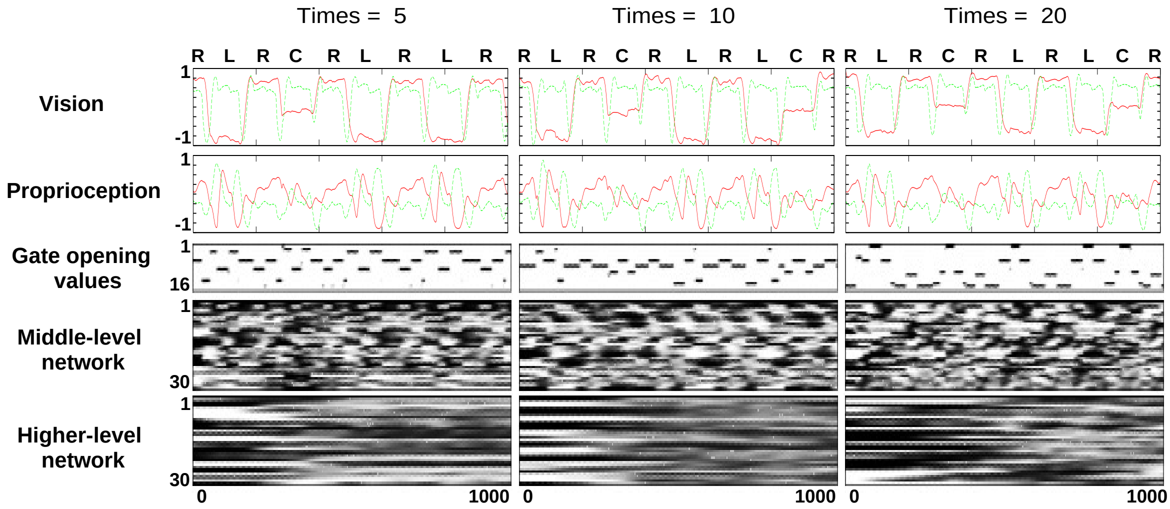

Figure 2. Time series generated by a trained network. This figure uses the same format as Figure 4 in the article, but shows transient dynamics starting from a specific initial state given by training. When times of transitions of primitive actions were 5 in training sets, the output was converged to a periodic movement such as "RLRLRL···" (where L and R are the left and right positions), and the neural activity in the higher-level network was also converged to a fixed point.
